# Supplementary material for: Homeostatic serum IgE is secreted by plasma cells in the thymus and enhances mast cell survival
Source: Nat Commun. 2022 Mar 17;13:1418. doi: 10.1038/s41467-022-29032-x (PMC8930980; doi:10.1038/s41467-022-29032-x)
Supplement: Supplementary file 1 — Supplementary Information [file 41467_2022_29032_MOESM1_ESM.pdf]

Supplementary Information

Homeostatic serum IgE is secreted by plasma cells in the thymus and enhances mast cell survival

Dong-il Kwon, Eun Seo Park, Mingyu Kim, Yoon Ha Choi, Myeong-seok Lee, Si-hyung Joo, Yeon-Woo Kang, Minji Lee, Saet-byeol Jo, Seung-Woo Lee, Jong Kyoung Kim and You Jeong Lee

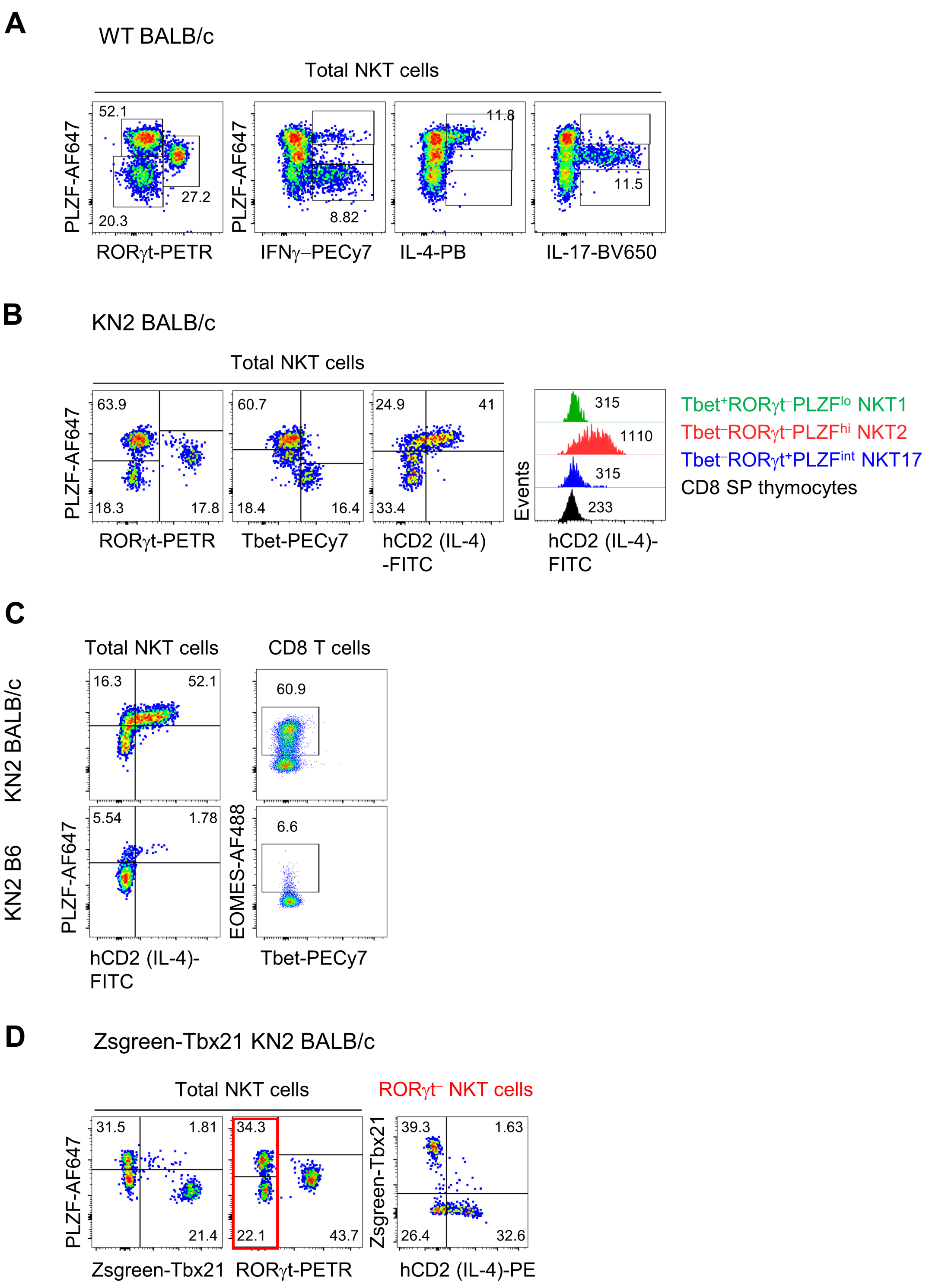

Supplementary Fig. 1. Tbet⁻RORγt⁻PLZF^{hi} NKT2 cells produce homeostatic IL-4 in the thymus. (A) Total thymocytes from 6-7 week-old WT BALB/c mice were stimulated with PMA and ionomycin and stained with indicated markers. Representative FACS plots show cytokine expression from iNKT cells. **(B)** Representative dot plots show expression of PLZF, RORγt, Tbet, and human CD2 (hCD2) in thymic NKT cells from 6-7 week-old KN2 BALB/c mice (left three panels). Histogram shows the MFI of hCD2 expression levels in each iNKT cell subset (right). **(C)** Representative dot plots of hCD2 expressing iNKT cells (left) and EOMES^{+} CD8 T cells (right) in the thymus of BALB/c and B6 mice were shown. **(D)** Representative FACS plots show hCD2 expression in Tbet^{-}RORγt^{-} thymic iNKT cells from Zsgreen-Tbx21 KN2 BALB/c mice. Numbers indicate frequencies of cells in adjacent gates. Representative results from more than three independent experiments were shown.

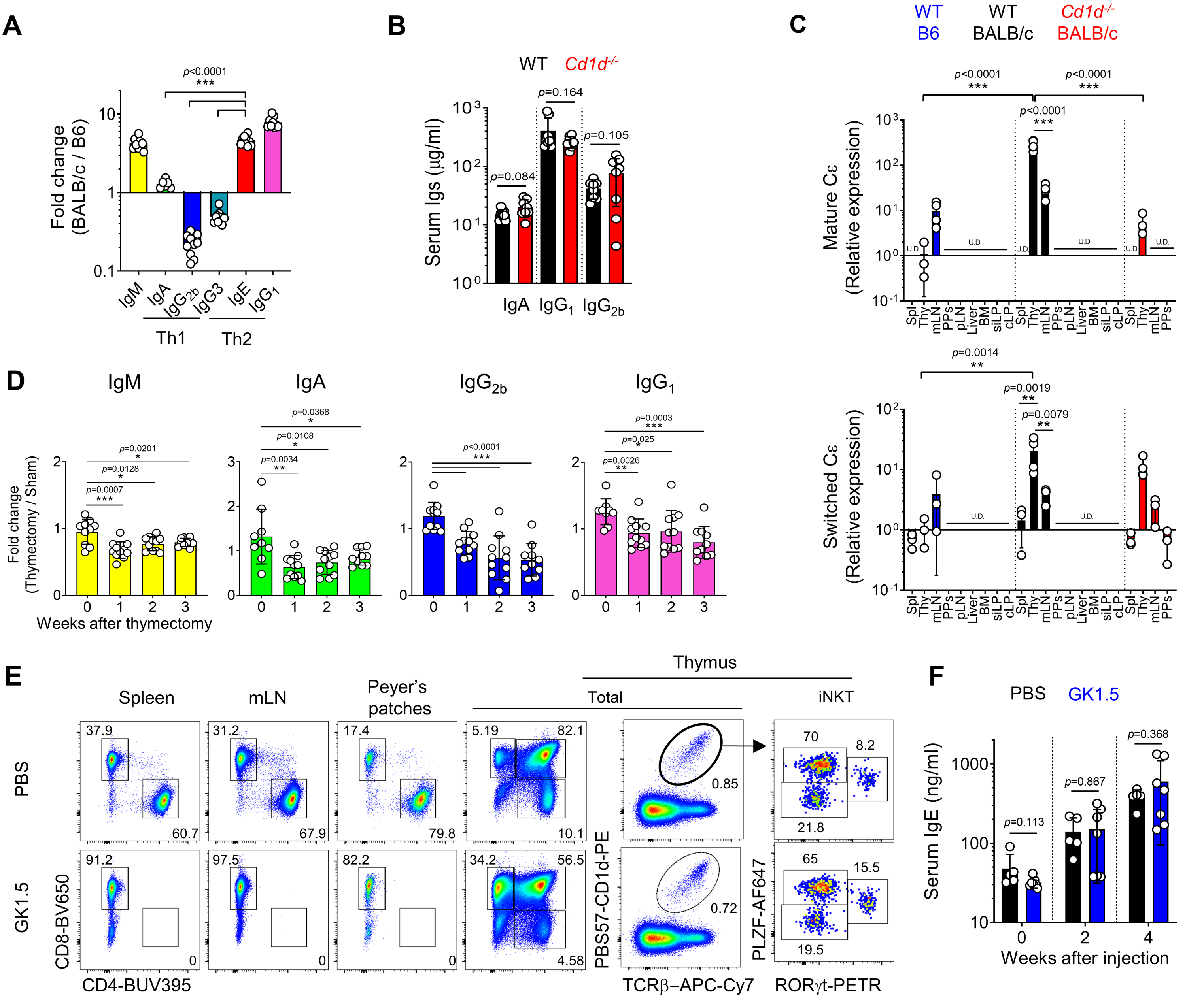

Supplementary Fig. 2. Thymus regulates serum immunoglobulin levels. (A) Serum immunoglobulin concentrations of B6 and BALB/c mice were measured by ProcartaPlex immunoassays, and graph shows their fold ratios (N=6-10). **(B)** Serum IgA, IgG1, and IgG2b concentrations from WT (N=8) and *Cd1d*$^{-/-}$ (N=8) BALB/c mice were measured by ELISA. **(C)** Graphs show relative mRNA expressions of switching (lower panel) transcripts of Cε from indicated lymphoid tissues of WT B6 (N=3) and BALB/c (N=4) mice mature (upper panel) and and *Cd1d*$^{-/-}$ BALB/c mice (N=3). The relative expression of transcripts was normalized to an average value obtained from the thymus of B6 mice. **(D)** Serum IgM, IgA, IgG1 and IgG2b levels were measured in thymectomized (N=10) or sham (N=5) operated BALB/c mice by ELISA and fold changes were calculated. **(E and F)** Peripheral CD4 T cells of BALB/c mice were depleted by injecting anti-CD4 antibody (GK1.5) every 3 days for 4 weeks from 2.5 week-old. Representative dot plots show presence of CD4 and NKT cells in indicated organs of PBS (N=5) or GK1.5 (N=7) treated BALB/c mice **(E)**. Numbers indicate frequencies of cells in adjacent gates. Total serum IgE concentrations of WT (N=5) and CD4-depleted mice (N=7) were measured by ELISA **(F)**. Data are presented as mean values ± SD (A, B, C, D, and F). Each dot represents an individual mouse. Unpaired two-tailed t-tests (A, B, D, F) and one-way ANOVA (C) were used for comparison. $^{***}p<0.001$, $^{**}p<0.01$, $^{*}p<0.05$. Not significant ($p>0.05$). U.D., undetected; Spl, spleen; Thy, thymus; mLN, mesenteric lymph node; PP, Peyer's patch; pLN, peripheral lymph node; BM, Bone marrow; siLP, small intestinal lamina propria; cLP, colonic lamina propria.

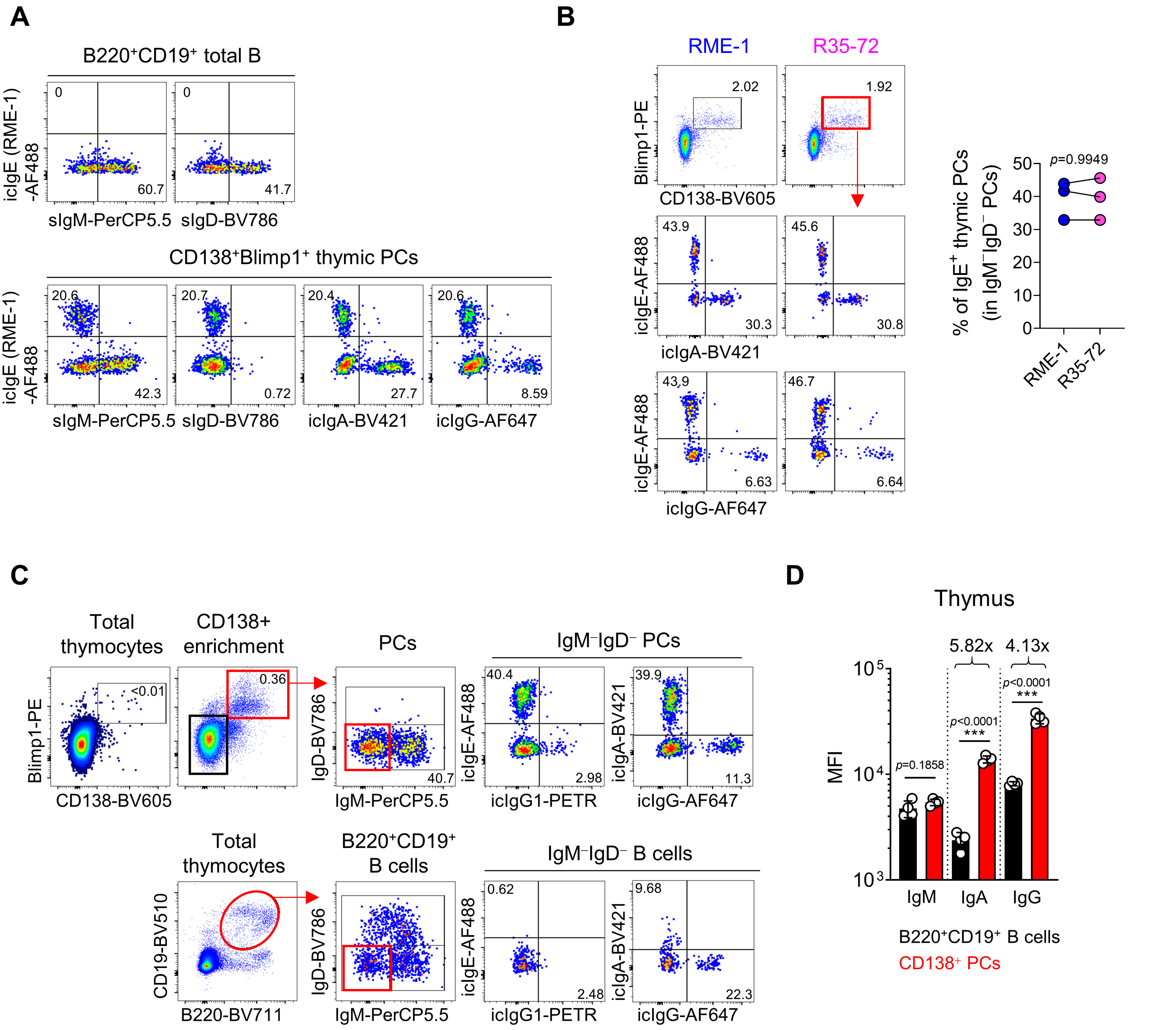

Supplementary Fig. 3. Thymic PCs express higher levels of intracellular immunoglobulins than B220⁺CD19⁺ B cells. (A) Representative FACS plots show staining of all Igs from B220⁺CD19⁺ B cells (upper) from total thymocytes and PCs (lower) after CD138 enrichment. **(B)** Total thymocytes from 6-7 week-old BALB/c were enriched for CD138⁺ PCs by MACS and then enriched cells were equally divided into two samples for staining different clone of anti-IgE antibody. Representative dot plots of IgE⁺ PCs using indicated clone of anti-IgE antibody are shown (N=3). **(C)** Single cell suspensions of BALB/c thymocytes were enriched for CD138⁺ cells by MACS and TCRβ⁺ cells were gated out. Representative dot plots show gating strategy for expression of intracellular (ic) IgE, IgA and IgG after enrichment of CD138⁺ cells (upper panels) or in total B220⁺CD19⁺ B cells (lower panels). **(D)** Graph shows MFI of indicated isotypes from CD138⁻B220⁺CD19⁺ follicular B cells and CD138⁺Blimp1⁺ PCs in BALB/c thymus (N=4). Data are presented as mean values ± SD. Unpaired two-tailed t-tests were used for comparison. Number indicate frequencies of cells in adjacent gates (A, B, C) and fold changes (D). *** p<0.001, $^{**}p$<0.01, $^{*}p$<0.05. Not significant (p>0.05). PC, plasma cell; FMO, fluorescence minus one; MFI, mean fluorescent intensity.

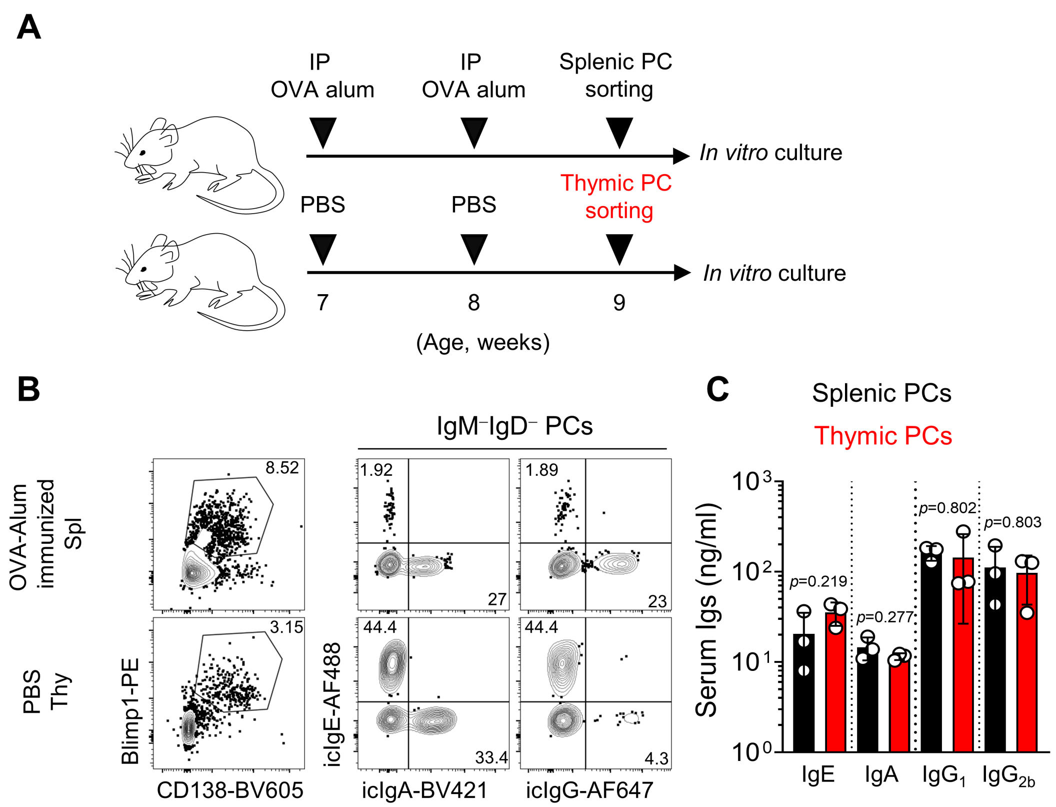

Supplementary Fig. 4. Immunogobulins secreted from thymic PCs is equivalent to those of canonical splenic PCs. (A) 7 week-old BALB/c mice were intraperitoneally injected with OVA-alum (upper) or PBS (lower) at day 0 and 7. At day 14, splenic and thymic PCs from pooled mice were sorted by FACS. Equal numbers of PCs were cultured for 5 days. **(B)** Total thymocytes and splenocytes from indicated mice were enriched for CD138$^+$ cells using MACS. Representative FACS plots show expression of intracellular immunoglobulins (icIgs) from indicated PCs before sorting. Numbers indicate frequencies of cells in adjacent gates. **(C)** Indicated cells were cultured for 5 days and total concentrations of IgE, IgA, IgG_1, and IgG_{2b} in supernatants were measured by ELISA (N=3). Concentrations were carlibrated by frequency of icIg$^+$ PCs. Results from three independent experiments were shown. Data are presented as mean values ± SD. Unpaired two-tailed *t*-test were used for comparison. Not significant (*p*>0.05).

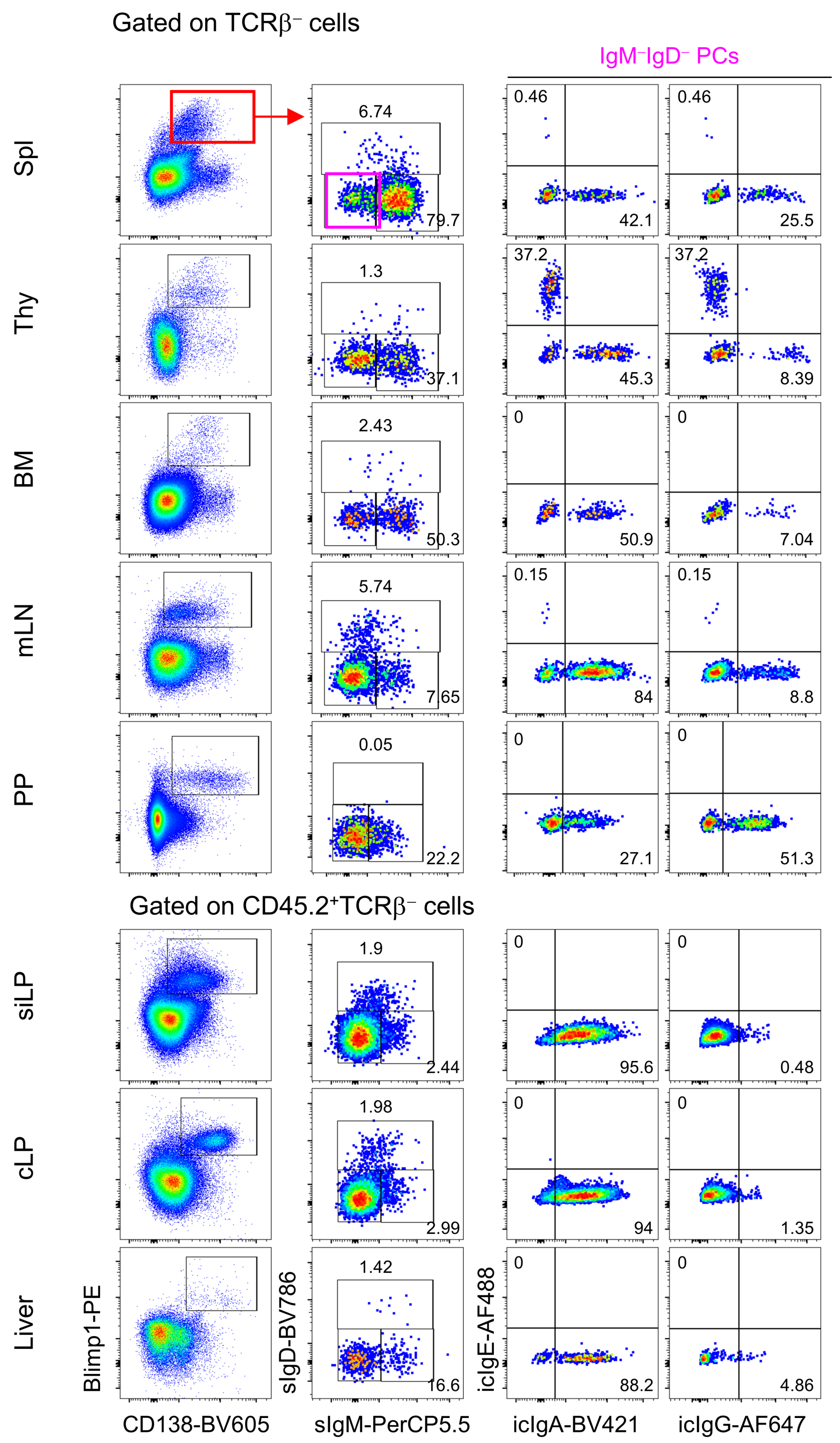

Supplementary Fig. 5. Immunoglobulin expression in plasma cells from various tissues. Single cell suspension from spleen, thymus, BM, and mLN was enriched for CD138⁺ PCs by using MACS. Enriched cells and total lymphocytes from PP, siLP, cLP, and liver were stained with indicated markers. Representativce FACS plots show PCs and their immunoglobulin (Ig) expression from indicated tissues. Numbers indicate frequencies of cells in adjacent gates. Spl, spleen; Thy, thymus; BM, bone marrow; mLN, mesenteric lymph node; PP, Peyer's patch; siLP, small intestinal lamina propria; cLP, clonic lamina propria.

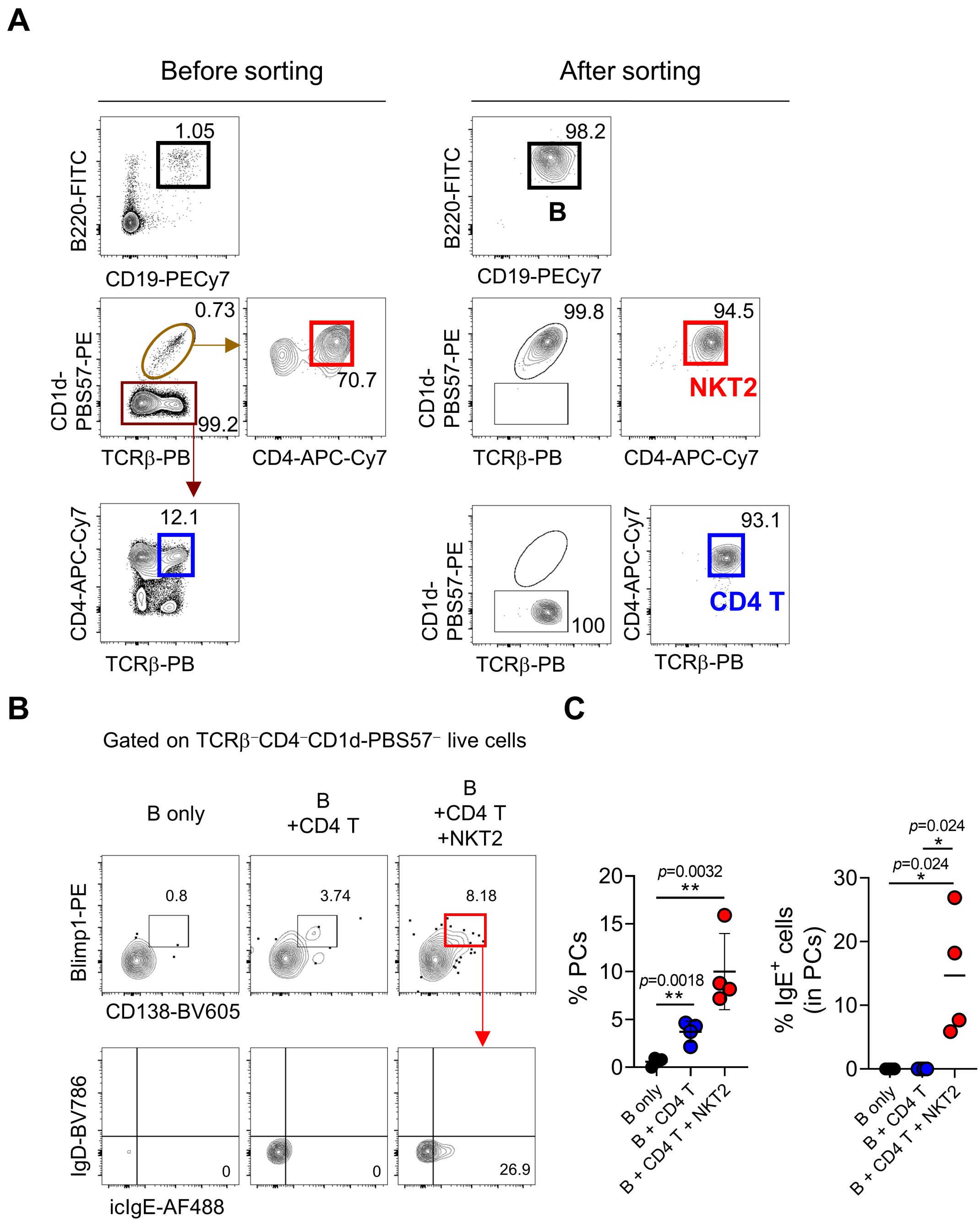

Supplementary Fig. 6. B220$^+$CD19$^+$ B cells are differentiated into PCs in FTOC experiments. B220$^+$CD19$^+$ B, NKT2 and CD4 T cells were sorted from adult thymi of BALB/c mice and cultured in FTOC for 7 days. (A) FACS plots show sorting scheme and purity. (B) Representative FACS plots shows the frequencies of PCs (upper) and IgE expression (lower). (C) Graph shows statistical analysis. Data are presented as mean values ± SD. Number indicates frequencies of cells in adjacent gates. Unpaired two-tailed *t*-test were used for comparison. *** $p<0.001$, $^{**}p<0.01$, $^{*}p<0.05$

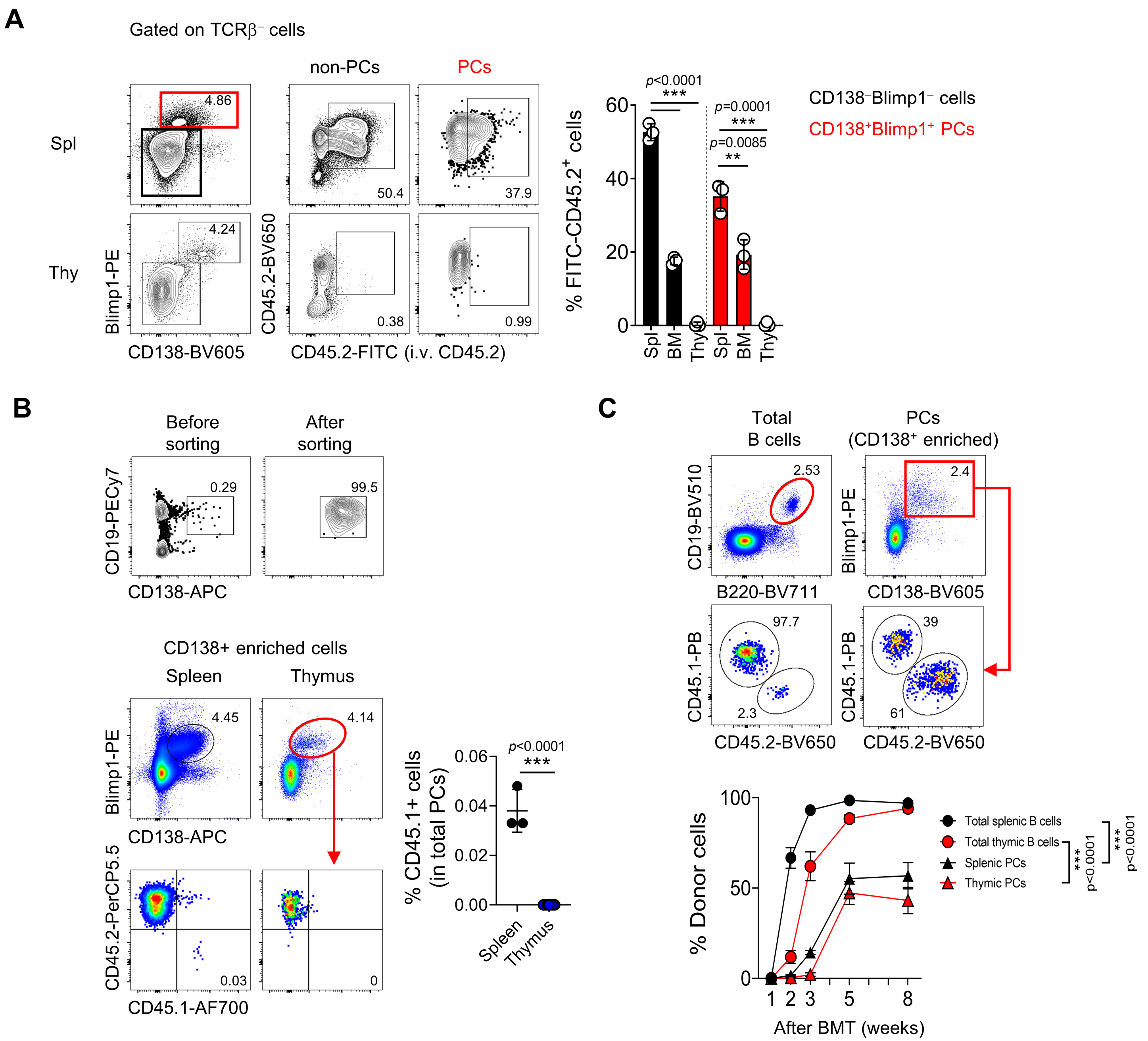

Supplementary Fig. 7. Thymic PCs are non-circulating BM derived cells. (A) FITC-conjugated CD45.2 antibodies were intravenously injected into BALB/c mice (N=3) and sacrificed three minutes later. Representative FACS plots show circulating PCs (left) and graph shows statistical analysis (right). **(B)** $CD19^+CD138^+$ PCs in spleen, mesenteric lymph node, Peyer's patches from CD45.1 mice were sorted and FACS plots show sorting purity (upper). Sorted PCs were transferred into CD45.2 host and total splenocytes and thymocytes were enriched for $CD138^+$ cells by MACS after 7 days. Representative dot plots show $CD45.1^+$ donor cells in the spleen (N=3) and thymus (N=7) of host mice and graph shows statistical analysis (lower) **(C)** $CD45.1^+$ bone marrow cells were transferred into lethally irradiated $CD45.2^+$ recipient BALB/c mice (N=3-6) and analysed at indicated points. Representative FACS plots show host- and donor-derived $B220^+CD19^+$ B cells (left) and plasma cells (right) in thymus (upper). Graph shows the kinetics of frequencies of donor-derived cells (lower). Numbers indicate frequencies of cells in adjacent gates. Data are presented as mean values ± SD (A, B, and C). Unpaired two-tailed t-tests (A and B) and two-way ANOVA (C) were used for comparison. *** p<0.001, $^{**}p$<0.01, $^{*}p$<0.05. PC, plasma cell; Spl, spleen; Thy, thymus; BM, bone marrow.

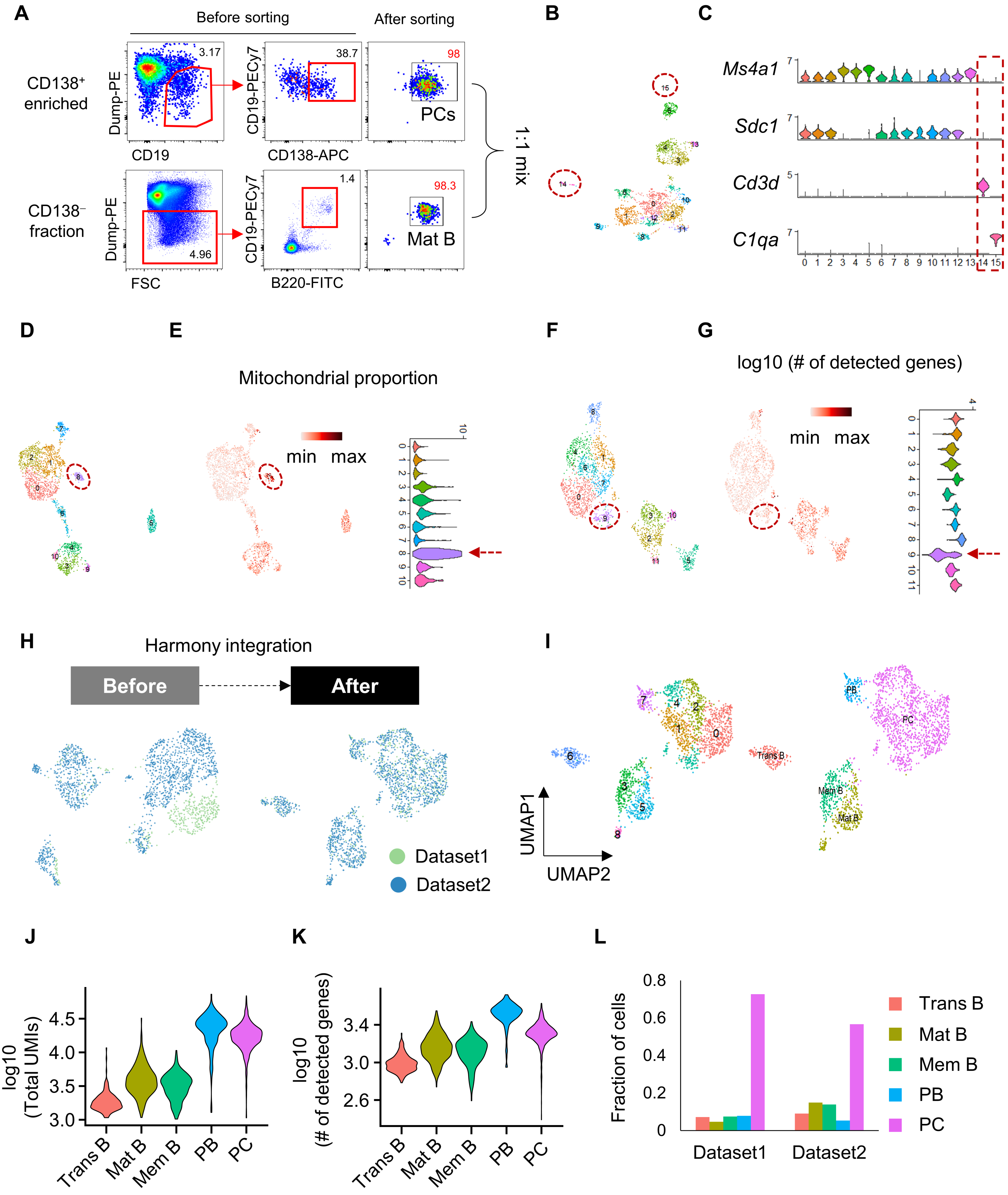
A
Before sorting
After sorting
CD138+ enriched
CD138– fraction
Dump-PE
CD19
CD19-PECy7
CD138-APC
FSC
B220-FITC
3.17
38.7
98
4.96
1.4
98.3
PCs
Mat B
1:1 mix
B
C
Ms4a1
Sdc1
Cd3d
C1qa
D
E
Mitochondrial proportion
min
max
F
G
log10 (# of detected genes)
H
Harmony integration
Before
After
Dataset1
Dataset2
I
UMAP1
UMAP2
PB
PC
Trans B
Mem B
Mat B
J
log10 (Total UMIs)
K
log10 (# of detected genes)
L
Fraction of cells
Trans B
Mat B
Mem B
PB
PC
Dataset1
Dataset2

Supplementary Fig. 8. Sorting purity and quality control for scRNA-seq. (A) Single cell suspensions of thymocytes from pooled thymi of 6-week-old BALB/c mice were enriched with $CD138^+$ cells by MACS. Enriched $CD138^+$ cells were FACS sorted into $CD19^+CD138^+$ PCs after gating out $dump^+$ ($CD3^+CD4^+CD11b^+$) cells (upper). $CD138^-$ flow through was sorted into $dump^-B220^+CD19^+$ B cells (lower). Representative FACS plots show purity of cells after sorting. **(B)** UMAP plot shows all cells coloured by clusters. **(C)** Violin plot shows the normalized expression levels of *Ms4a1*, *Sdc1*, *Cd3d*, *C1qa*. **(D)** UMAP plot show cells after removing cluster 14, 15 from (A), which are coloured by clusters. **(E)** UMAP plot (left) and violin plot (right) show the percentage of UMIs assigned to mitochondrial genes per cell for each cluster. **(F)** UMAP plot show cells after removing cluster 9 from (D), which are coloured by clusters. **(G)** UMAP plot (left) and violin plot (right) show the number of detected genes per cell for each cluster. **(H)** UMAP plots shows cells coloured by pooled replicates before and after batch correction. **(I)** UMAP plot shows QC-positive cells coloured by clusters (left) and annotated B cell subtype (right). **(J)** Violin plot shows the number of UMIs per cell for each annotated B cell subtype. **(K)** Violin plot shows the number of detected genes per cell for each annotated B cell subtypes. **(L)** Bar plot shows fractions of cells between two pooled replicates for each annotated B cell subtype. Trans B, transitional B cell; Mat B, mature B cell; Mem B, memory B cell; PB, plasmablast; PC, plasma cell.

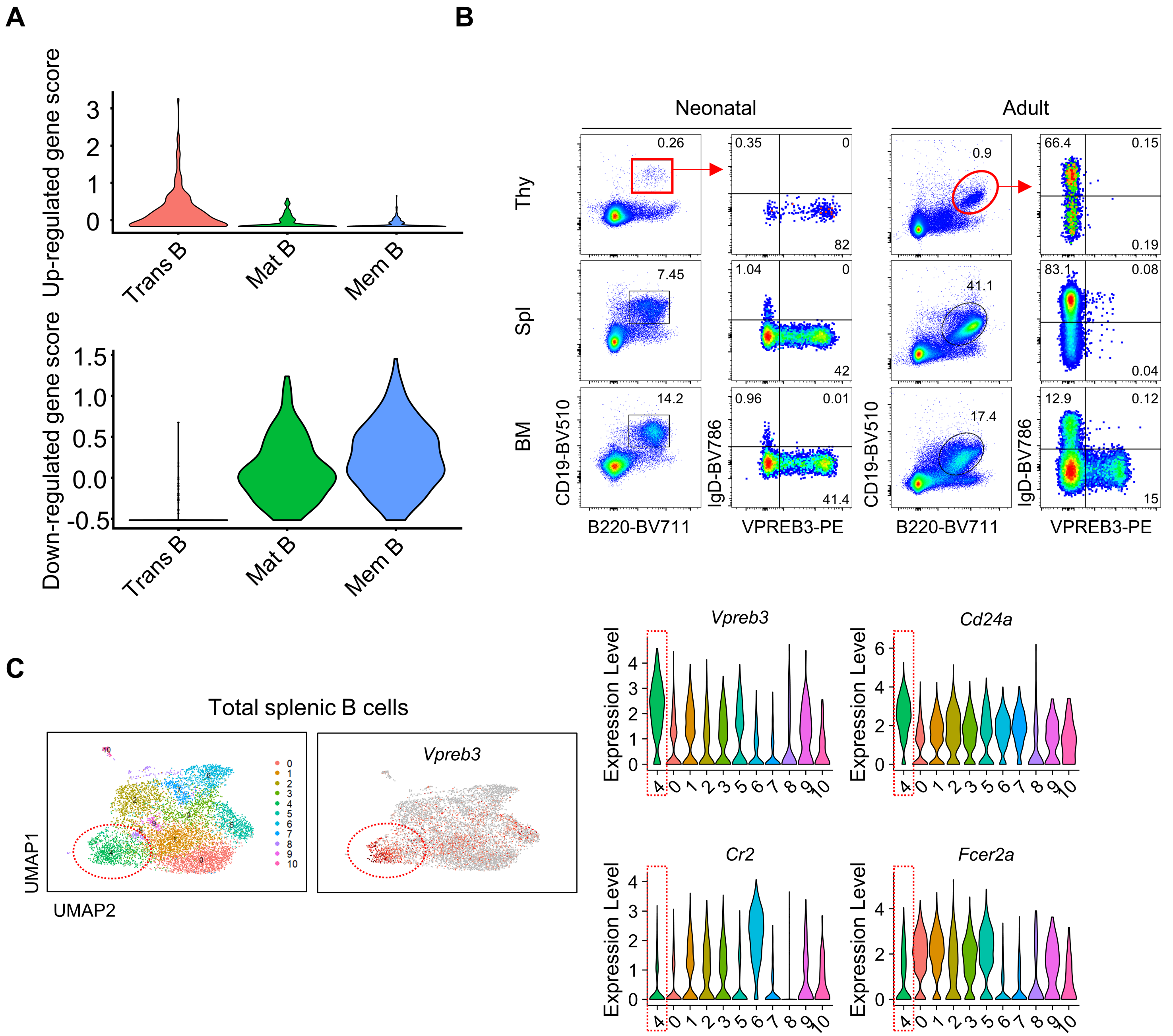

Supplementary Fig. 9. Transitional B cells transcriptionally express Vpreb3. (A) Violin plots show enrichment score of transitional B cell signature genes obtained from Kleiman, E. *et al.* (Ref #26) in indicated clusters. (B) Representative FACS plots show VPREB3 expression in total B cells from thymus, spleen, and BM of neonatal and adult BALB/c mice. Numbers indicate frequencies of cells in adjacent gates. (C) UMAP clusters and *Vpreb3* expression in splenic B cells were shown using a public scRNA-seq dataset (left). Violin plots show expression levels of *Vpreb3, Cd24a*, *Cr2*, *Fcer2a* in each cluster (right). Thy, thymus; Spl, spleen.

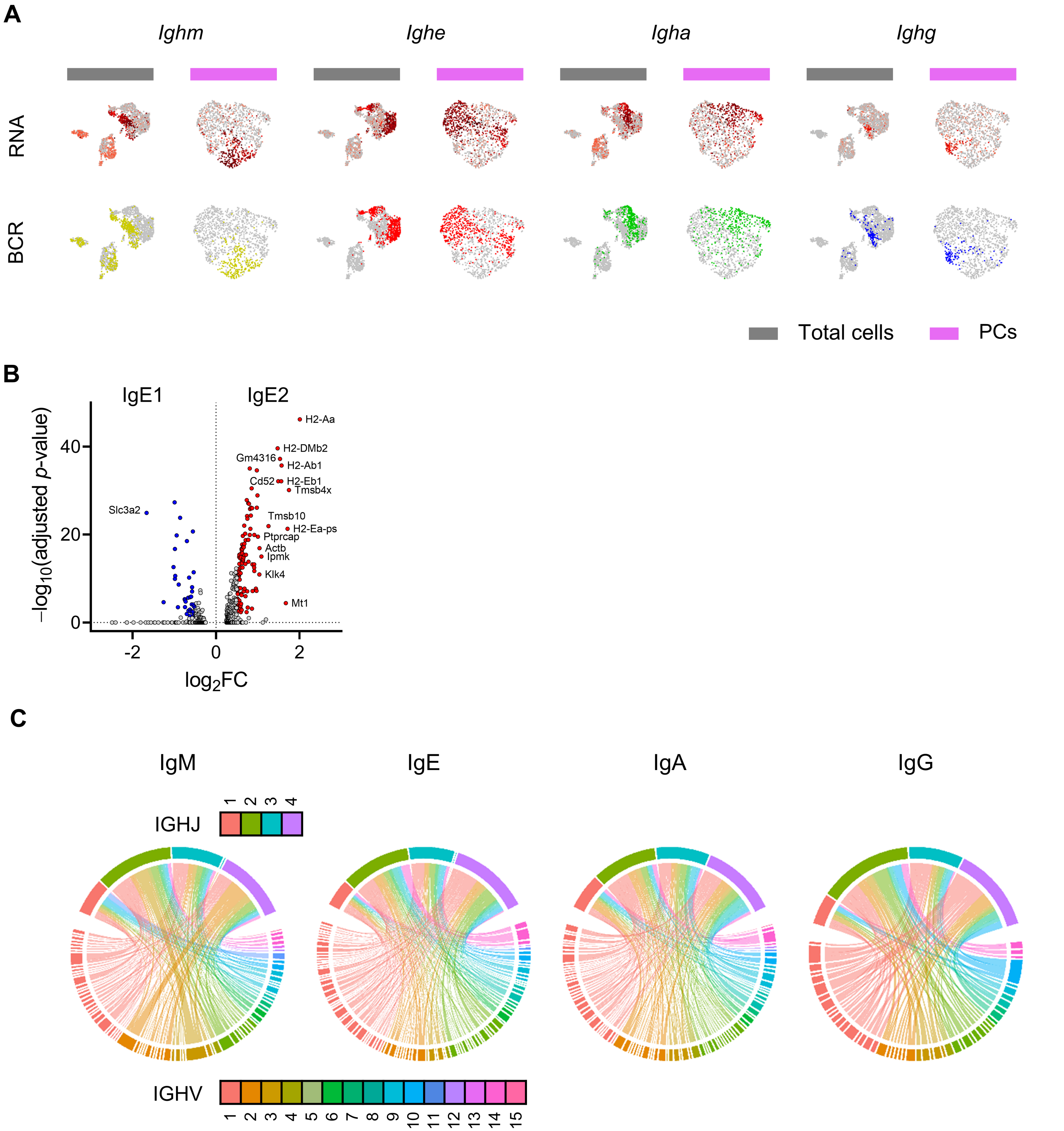

Supplementary Fig. 10. BCR repertoire analysis. (A) UMAP plots show cells coloured by expression levels of each IGHC coding gene (top) and isotype obtained from paired V(D)J sequencing (bottom). **(B)** Volcano plot of differentially expressed genes between IgE2 and IgE1 subtype. **(C)** Circos plots show heavy chain V-J gene pair per isotype. PC, plasma cell.

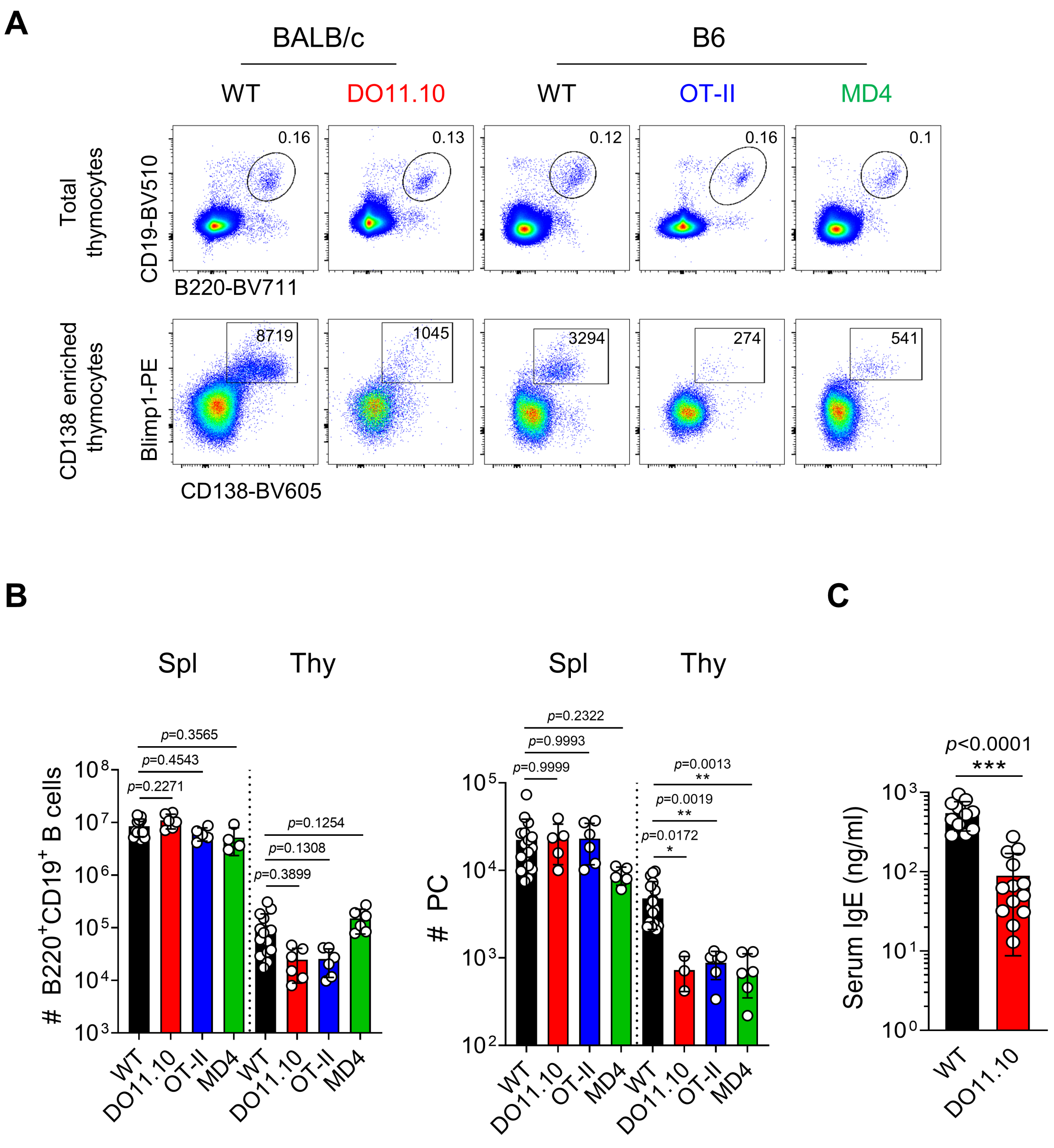

Supplementary Fig. 11. Development of thymic PCs is inhibited in DO11.10, OT-II and MD4 Tg mice. (A) Representative dot plots show thymic B220⁺CD19⁺ B cells and CD138⁺Blimp1⁺ PCs from WT (N=13-17), OT-II (N=6), DO11.10 (N=3-6) and MD4 (N=5-6) mice. **(B)** Graphs show their numbers with statistical analysis. Pooled results are from 3 independent experiments. Numbers indicate cell numbers or frequencies in adjacent gates. **(C)** Serum levels of IgE from WT (N=11) and DO11.10 (N=13) mice were measured by ELISA. Data are presented as mean ± SD (B and C). Each dot represents an individual mouse. One-way ANOVA (B) and unpaired two-tailed t-tests (C) were used for comparison. *** p<0.001, $^{**}p$<0.01, $^{*}p$<0.05. Not significant (p>0.05). Spl, spleen; Thy, thymus.

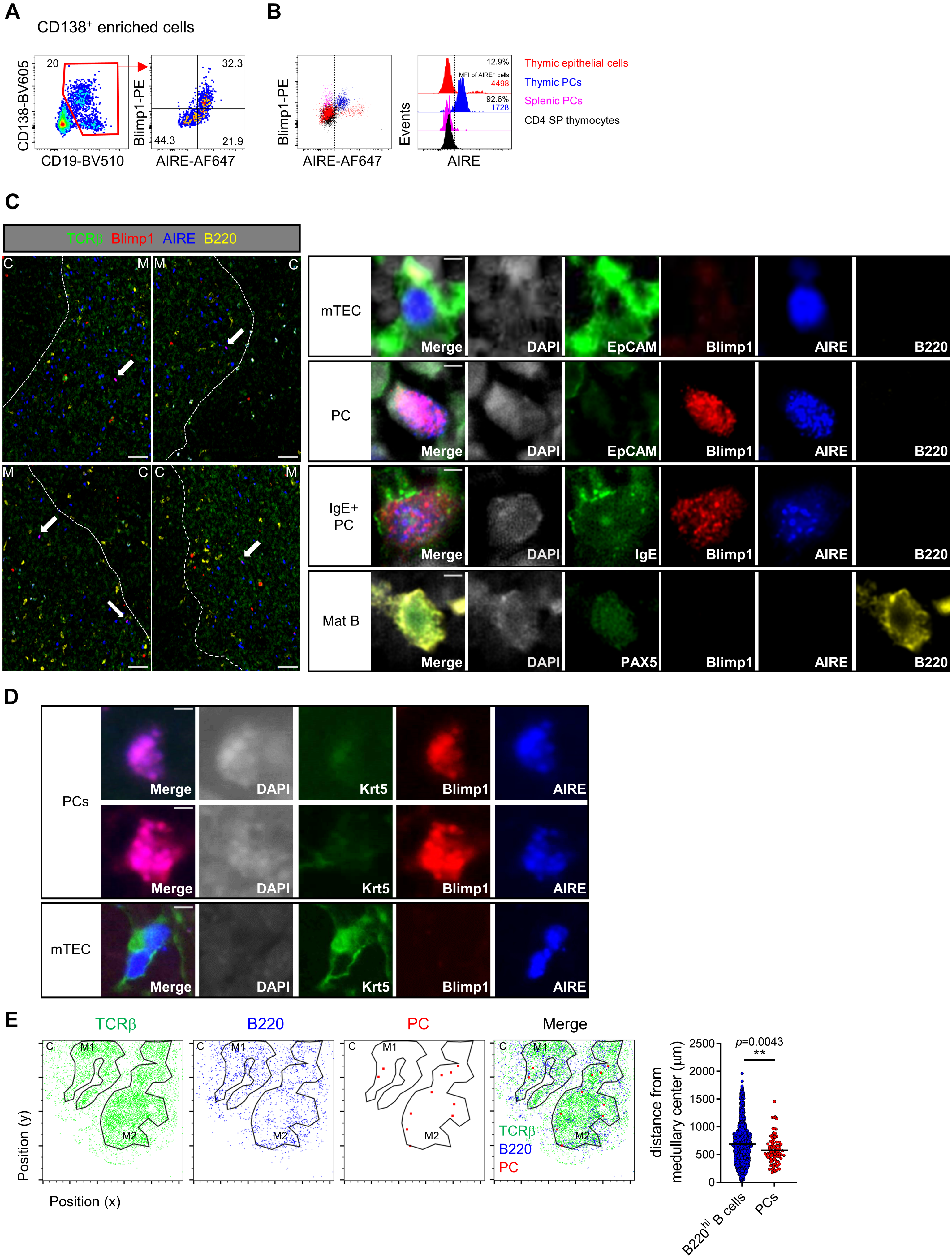

A
CD138+ enriched cells
20
CD138-BV605
CD19-BV510
32.3
44.3
21.9
Blimp1-PE
AIRE-AF647
B
Blimp1-PE
AIRE-AF647
Events
AIRE
12.9%
MFI of AIRE+ cells
4498
92.6%
1728
Thymic epithelial cells
Thymic PCs
Splenic PCs
CD4 SP thymocytes
C
TCRβ Blimp1 AIRE B220
C
M
mTEC
PC
IgE+ PC
Mat B
Merge
DAPI
EpCAM
IgE
PAX5
Blimp1
AIRE
B220
D
PCs
mTEC
Merge
DAPI
Krt5
Blimp1
AIRE
E
TCRβ
B220
PC
Merge
M1
M2
Position (y)
Position (x)
p=0.0043
**
distance from medullary center (µm)
2500
2000
1500
1000
500
0
B220hi B cells
PCs

Supplementary Fig. 12. Thymic PCs express AIRE and locate in the medulla. **(A and B)** Total thymocytes of BALB/c mice were digested with enzymes to isolate epithelial cells or enriched for $CD138^+$ cells using MACS and stained with indicated markers. Representative dot plots of $AIRE^+Blimp1^+$ cells were shown after gating out $CD4^+$ thymocytes (A). Representative FACS plots (left) and histogram (right) show AIRE expression from indicated cell types (B). Numbers indicate frequencies of cells in adjacent gates or MFI values. **(C and D)** Thymi from WT BALB/c mice were stained with indicated markers and imaged Scale bars represent 50 μm (C, left) and 2 μm (C, right and D) **(E)** Medullary regions (M1 and M2) were identified by density gradient of TCRβ staining, and localization of $B220^{hi}$ B cells and $AIRE^+Blimp1^+$ PCs were analyzed by histocytometry (left four panels). Graph shows the distance of each cell from medullary centers (right). Each dot represents a single cell. Representative results from four independent experiments are shown. Unpaired two-tailed t-tests were used for comparison. $^{**}p<0.01$. Mat B, mature B cells; PC, plasma cell; SP, single positive; C, cortex; M, medulla.

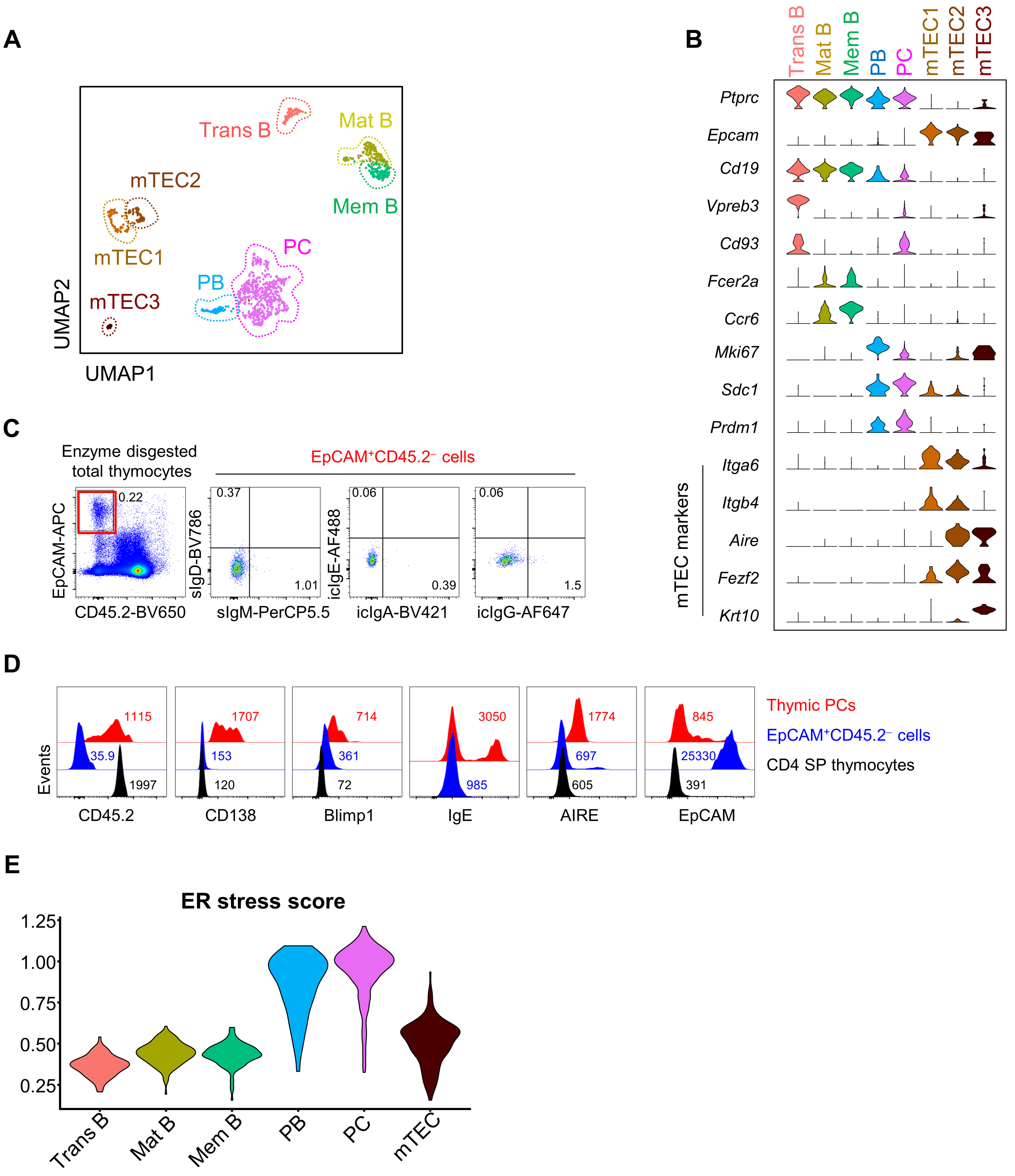

Supplementary Fig. 13. Thymic plasma cells are distinct population from mTEC. (A) UMAP plots show all cells and annotated B cell subsets and mTECs were labeld with indicated colors. **(B)** Violin plots show the gene expression of B cell and mTEC markers. **(C and D)** Single cell suspenstions of enzyme digested thymus of 6-7 week-old BALB/c mice were stained with indicated markers. Representative FACS plots show expression of all immunoglobulins (C). Histogram shows MFI of CD45.2, CD138, Blimp1, IgE, AIRE, and EpCAM expression in indicated cells (D). **(E)** Violin plot shows stress score of endoplastic reticulum (ER) from indicated cells. Number indicate frequencies of cells adjacent gates (C) or MFI value (D).

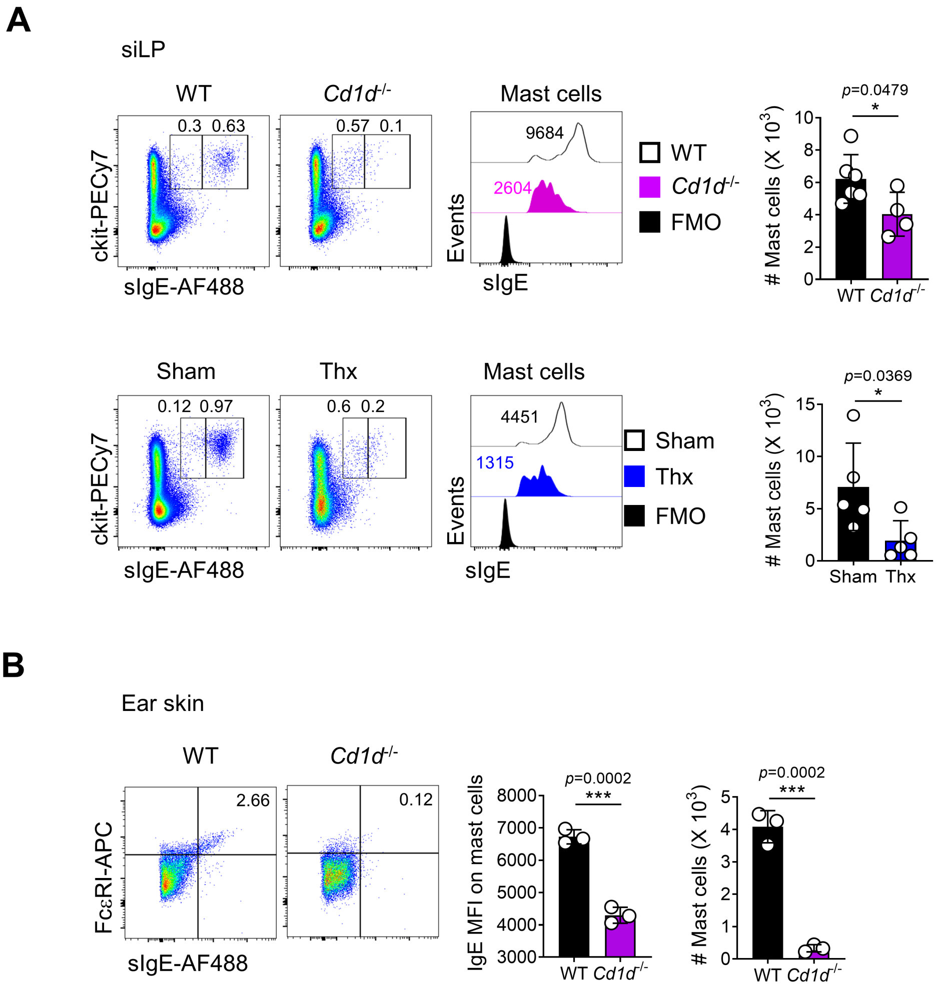

Supplementary Fig. 14. iNKT cells regulate mast cell homeostasis in the gut and ear skin. (A) Representative FACS plots show $CD45^{+}TCRb^{-}CD19^{-}CD11b^{-}Siglef^{-}ckit^{+}Fc\varepsilon RI^{+}sIgE^{+}$ mast cells in siLP of *Cd1d*$^{-/-}$ and thymectomized (Thx) BALB/c mice, and histograms show surface IgE levels on mast cells. Graphs show statistical analysis of their numbers (right, N=3-5). **(B)** Representative dot plots show $CD45^{+}TCR\beta^{-}CD19^{-}CD49b^{-}Fc\varepsilon RI^{+}sIgE^{+}$ mast cells in ear skin with statistical analysis. Graph shows MFI of IgE on skin mast cells and their numbers from indicated mice. Numbers indicate frequencies of cells in adjacent gates. Data are presented as mean values ± SD (A and B). Each dot represents an individual mouse. Unpaired two-tailed t-tests were used for comparison (A and B). *** p<0.001, **p<0.01, *p<0.05. siLP, small intestinal lamina propria; MFI, mean fluorescent intensity

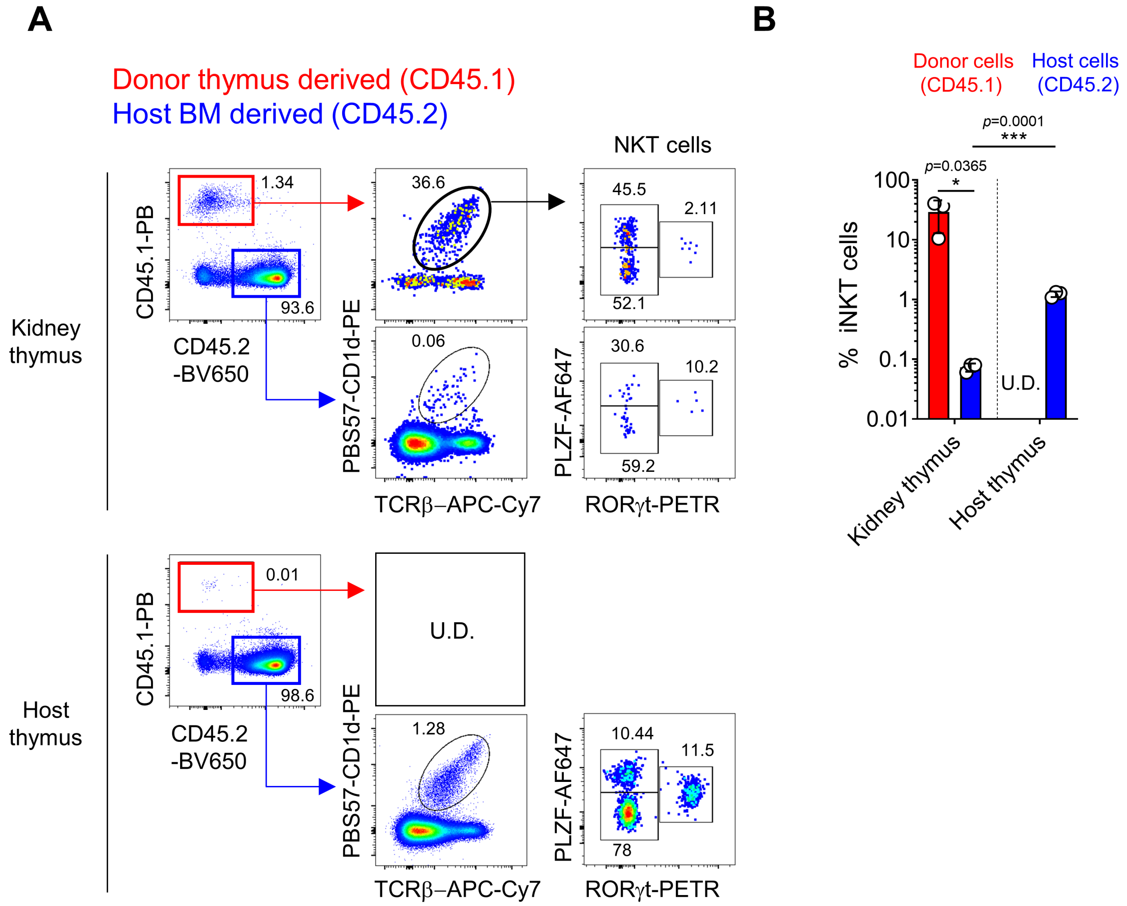

Supplementary Fig. 15. Adult BM derived precursors are inefficient in generating thymic iNKT cells. $CD45.2^+$ BALB/c mice (N=3) were transplanted with thymic lobes from $CD45.1^+$ newborn mice under the kidney capsules. After 4 weeks, transplanted and host thymi were analyzed. **(A)** Representative dot plots show the frequency of total NKT cells and their subsets among $CD45.1^+$ donor (red) and $CD45.2^+$ host-derived (blue) cells from transplanted kidney thymus (upper) and host neck thymus (lower). Numbers indicate frequencies of cells in adjacent gates. **(B)** Graph shows frequencies of iNKT cells in indicated cells. Data are presented as mean values ± SD. Each dot represents an individual mouse. Unpaired two-tailed t-tests were used for comparison. $^{***}p<0.001$, $^{**}p<0.01$, $^{*}p<0.05$. U.D., undetected.
