# Supplementary material for: Homeostatic serum IgE is secreted by plasma cells in the thymus and enhances mast cell survival
Source: Nat Commun. 2022 Mar 17;13:1418. doi: 10.1038/s41467-022-29032-x (PMC8930980; doi:10.1038/s41467-022-29032-x)
Supplement: Supplementary file 3 — Reporting summary [file 41467_2022_29032_MOESM3_ESM.pdf]

nature portfolio

Corresponding author(s): Jong Kyoung Kim
You Jeong Lee

Last updated by author(s): Nov 26, 2021

Reporting Summary

Nature Portfolio wishes to improve the reproducibility of the work that we publish. This form provides structure for consistency and transparency in reporting. For further information on Nature Portfolio policies, see our Editorial Policies and the Editorial Policy Checklist.

Statistics

For all statistical analyses, confirm that the following items are present in the figure legend, table legend, main text, or Methods section.

n/a	Confirmed	
☐	☒	The exact sample size (n) for each experimental group/condition, given as a discrete number and unit of measurement
☐	☒	A statement on whether measurements were taken from distinct samples or whether the same sample was measured repeatedly
☐	☒	The statistical test(s) used AND whether they are one- or two-sided<br>*Only common tests should be described solely by name; describe more complex techniques in the Methods section.*
☒	☐	A description of all covariates tested
☒	☐	A description of any assumptions or corrections, such as tests of normality and adjustment for multiple comparisons
☐	☒	A full description of the statistical parameters including central tendency (e.g. means) or other basic estimates (e.g. regression coefficient) AND variation (e.g. standard deviation) or associated estimates of uncertainty (e.g. confidence intervals)
☐	☒	For null hypothesis testing, the test statistic (e.g. F, t, r) with confidence intervals, effect sizes, degrees of freedom and P value noted<br>*Give P values as exact values whenever suitable.*
☒	☐	For Bayesian analysis, information on the choice of priors and Markov chain Monte Carlo settings
☒	☐	For hierarchical and complex designs, identification of the appropriate level for tests and full reporting of outcomes
☒	☐	Estimates of effect sizes (e.g. Cohen's d, Pearson's r), indicating how they were calculated

Our web collection on statistics for biologists contains articles on many of the points above.

Software and code

Policy information about availability of computer code

Data collection	Single-cell RNA sequencing data and BCR sequencing data collection:<br>CellRanger v3.1.0, DropletUtils (v1.61) R package, Scater (v1.15.6) R package, Scran (v1.14.6) R package, Seurat (v3.1.5) R package, Harmony (v1.0) R package, Change-O (v1.0.0) python package, Alakazam (v1.0.2) R package, WebLogo (v3.7.4), Peptides (v2.4.2) R package, Circlize (v0.4.11) R package
Data analysis	Flowjo V10. GaphPad Prism v7/v9

For manuscripts utilizing custom algorithms or software that are central to the research but not yet described in published literature, software must be made available to editors and reviewers. We strongly encourage code deposition in a community repository (e.g. GitHub). See the Nature Portfolio guidelines for submitting code & software for further information.

Data

Policy information about availability of data

All manuscripts must include a data availability statement. This statement should provide the following information, where applicable:

- Accession codes, unique identifiers, or web links for publicly available datasets
- A description of any restrictions on data availability
- For clinical datasets or third party data, please ensure that the statement adheres to our policy

The authors declare that all data that support our findings in this study are included in the supplemental information and available upon reasonable requests to the corresponding authors. Source Data are provided with this paper. The single-cell RNA and BCR sequencing data in this study have been deposited in the SRA database under the accession code PRJNA681739 ["https://www.ncbi.nlm.nih.gov/bioproject/PRJNA681739"] and are available. The processed single cell data and source data are available at https://github.com/CB-postech/NATURE-COMMUNICATIONS-thymus-plasmcells.

Field-specific reporting

Please select the one below that is the best fit for your research. If you are not sure, read the appropriate sections before making your selection.

- [x] Life sciences
- [ ] Behavioural & social sciences
- [ ] Ecological, evolutionary & environmental sciences

For a reference copy of the document with all sections, see nature.com/documents/nr-reporting-summary-flat.pdf

Life sciences study design

All studies must disclose on these points even when the disclosure is negative.

Sample size	We usually used 2-4 mice for each experiment with more than 2 times replication. We found that this sample size is sufficient to conclude significant difference among experimental groups.
Data exclusions	No data were excluded.
Replication	All experiments were performed at least twice and repeated results were successfully obtained. Details are shown in the figure legends.
Randomization	We matched all age and sex for each experimental group for in vivo experiments. Experimental animals were randomly allocated.<br>For single-cell RNA sequencing experiments, we used eighteen 6 week-old female mice.
Blinding	Blinding assessment was performed during food anaphylaxis experiments. However, mouse genotypes were not blinded to investigators prior to data acquisition.

Reporting for specific materials, systems and methods

We require information from authors about some types of materials, experimental systems and methods used in many studies. Here, indicate whether each material, system or method listed is relevant to your study. If you are not sure if a list item applies to your research, read the appropriate section before selecting a response.

Materials & experimental systems

n/a	Involved in the study	
[ ]	[x]	Antibodies
[x]	[ ]	Eukaryotic cell lines
[x]	[ ]	Palaeontology and archaeology
[ ]	[x]	Animals and other organisms
[x]	[ ]	Human research participants
[x]	[ ]	Clinical data
[x]	[ ]	Dual use research of concern

Methods

n/a	Involved in the study	
[x]	[ ]	ChIP-seq
[ ]	[x]	Flow cytometry
[x]	[ ]	MRI-based neuroimaging

Antibodies

Antibodies used

All antibodies were used for mouse samples
Anti-CD3e PE (Invitrogen, clone 145-2C11, #12-0031-83, 1:200)
Anti-CD4 BUV395 (BD, clone GK1.5, #563790, 1:400)
Anti-CD4 PE (Invitrogen, clone RM4-5, #12-0042-82, 1:400)
Anti-CD4 APC-Cy7 (Invitrogen, clone RM4-5, #47-0042-82, 1:200)
Anti-CD5 PerCP5.5 (Biolegend, clone 53-7.3, #100616, 1:400)
Anti-CD8a BV650 (BD, clone 53-6.7 #563234, 1:400)
Anti-CD11b PE (eBioscience, clone M1/70, #12-0112-82, 1:400)
Anti-CD11b PerCP5.5 (BD, clone M1/70, # 550993, 1:1000)
Anti-CD19 BV510 (BD, clone 1D3, #562956, 1:200)
Anti-CD19 PE-Cy7 (BD, clone 1D3, #5528541, 1:200)
Anti-CD21 PE-Cy7 (eBioscience, clone 8D9, #25-0211-82, 1:400)
Anti-CD23 FITC (eBioscience, clone B3B4, #11-0232-82, 1:400)
Anti-CD24 BV605 (Biolegend, clone M1/69, #101827, 1:1000)
Anti-CD38 AF700 (Invitrogen, clone 90, #56-0381-82, 1:400)
Anti-CD43 PE-Cy7 (Biolegend, clone 1B11, #121218, 1:1000)
Anti-CD44 redFluor710 (TONBO, clone IM7, #80-0441-U100, 1:400)
Anti-CD45.1 PB (Biolegend, clone A20, #110722, 1:200)
Anti-CD45.1 AF700 (BD, clone A20, #561235, 1:200)
Anti-CD45.2 BV650 (Biolegend, clone 104, #109836, 1:200)

Anti-CD45.2 PerCP5.5 (Invitrogen, clone 104, #45-0454-82, 1:200)
Anti-CD45.2 FITC (Invitrogen, clone 104, #11-0454-82, 1:200)
Anti-CD45R/B220 BV711(BD, clone RA3-6B2, #563892, 1:1000)
Anti-CD45R/B220 APC (Invitrogen, clone RA3-6B2, #17-0452-82, 1:400)
Anti-CD45R/B220 FITC (TONBO, clone RA3-6B2, 1:400)
Anti-CD45R/B220 BV480 (BD, clone RA3-6B2, #565631, 1:200)
Anti-CD49b PB (Biolegend, clone DX5, #108918, 1:200)
Anti-CD80 PE (eBioscience, clone 16-10A1, #12-0801-82, 1:100)
Anti-CD86 FITC (eBioscience, clone GL1, #11-0826-82, 1:100)
Anti-CD93 APC (eBioscience, clone AA4.1, #17-5892-81, 1:200)
Anti-CD117 (ckit) PE-Cy7 (Invitrogen, clone 2B8, #25-1171-82, 1:1000)
Anti-CD138 BV605 (BD, clone 281-2, #563147, 1:200)
Anti-CD138 Biotin (BD, clone 281-2, #553713, 1:200)
Anti-CD170 (Siglec-f) PE (BD, clone E50-2440, #552126, 1:1000)
Anti-CD183 (CXCR3) PE-Cy7 (Biolegend, clone CXCR3-173, #126516, 1:100)
Anti-CD185 (CXCR5) BV421 (BD, clone 2G8, #562889, 1:50)
Anti-CD196 (CCR6) BV421 (Biolegend, clone 29-2L17, #129828, 1:200)
Anti-CD326 (EpCAM) FITC (Biolegend, clone G8.8, #118207, 1:200)
Anti-TCR beta chain APC-Cy7 (BD, clone H57-597, #560656, 1:200)
Anti-FceRI APC (Invitrogen, clone 01-Mar, #17-5898-82, 1:100)
Anti-MHC-II APC (eBiosciecne, clone M5/114.15.2, #17-5321-82, 1:4000)
Anti-Ly6C eFlour450 (Invitrogen, clone HK1.4, #48-5932-82, 1:400)
Anti-VPREB3 PE (Biolegend, clone R3, #143604, 1:100)
Anti-EOMES eFluor 450 (Thermo Fisher Scientific, clone Dan11mag, #48-4875-82, 1:100)
Anti-EOMES AF488 (eBiosciecne, clone Dan11mag, #53-4875-82, 1:200)
Anti-IFN-gamma PE-CF594 (BD, clone XMG1.2, #562303, 1:400)
Anti-IL-4 BV421 (Biolegend, clone 11B11, #504119, 1:100)
Anti-Ki67 FITC (Thermo Fisher Scientific, clone SolA15, #11-5698-82, 1:200)
Anti-IL-17A BV650 (BD, clone TC11-18H10, #564170, 1:400)
Anti-PLZF AF647 (BD, clone R17-809, #563490, 1:400)
Anti-RORgt PerCP5.5 (BD, clone Q31-378, #562683, 1:400)
Anti-RORgt PETR (BD, clone Q31-378, #562684, 1:400)
Anti-Tbet PE-Cy7 (Thermo Fisher Scientific, clone eBio4B10, #25-5825-82, 1:400)
Anti-Blimp1 PE (Invitrogen, clone 5E7, #12-9850-82, 1:100)
Anti-PAX5 APC (eBioscience, clone 1H9, #17-9918-80, 1:400)
Anti-PAX5 AF488 (BD, Clone 1H9, #562816, 1:200)
Anti-IRF4 PE (eBioscience, clone 3E4, #12-9858-82, 1:200)
Anti-IgM PerCP5.5 (BD, clone R6-60.2, #550881, 1:200)
Anti-IgD BV786 (BD, clone 11-26c.2a, #563618, 1:2000)
Anti-IgA BV421 (BD, clone C10-1, #743293, 1:400 for surface staining and 1:4000 for intracellular staining)
Anti-IgE AF488 (Biolegend, clone RME-1, #406910, 1:400 for surface staining and 1:4000 for intracellular staining)
Anti-IgE FITC (BD, clone R35-72, #553415, 1:400 for surface staining and 1:4000 for intracellular staining)
Anti-IgG(Fc) AF647 (SouthernBiotech, polyclonal, #1033-31, 1:400 for surface staining and 1:10000 for intracellular staining)
Anti-IgG1 PETR (Jackson ImmunoResearch, polyclonal, #115-585-205, 1:1000)
Anti-AIRE eFluor660 (Invitrogen, clone 5H12, #50-5934-82, 1:400)
Anti-Cytokeratin5 (Abcam, polyclonal, #ab53121, 1:400)

Human antibody:

Anti-human CD2 FITC (BD, clone RPA-2.10, #555326, 1:50)

Secondary antibodies:

Streptavidin PE (BD Pharmingen, #554061) is used for CD1d tetramer
Anti-Rabbit IgG AF488 (Biolegend, Poly4064, #406416, 1:1000)
Anti-Rabbit IgG AF555 (Invitrogen, polyclonal, #A32732, 1:1000)

Other:
Anti-PE antibody (Novusbiologicals, polyclonal, NB120-7011, 1:200)
Streptavidin MicroBeads (Miltenyi Biotec, #130-048-101, 1:25)
PBS-57 loaded CD1d monomers were obtained from the tetramer facility of the US National Institutes of Health.

Validation

All antibodies are commercially available and have been routinely validated by manufacturers as described on their websites. All antibodies were tested in the laboratory using positive and negative controls and titrated before all experiments. FMO (Fluorescence Minus One) or isotype control staining was used to determine positive staining.

Animals and other organisms

Policy information about studies involving animals; ARRIVE guidelines recommended for reporting animal research

Laboratory animals	All the mice are maintained in the animal facility of Pohang University of Science and Technology (POSTECH) Biotech Center in accordance with the Institutional Animal Care and Use Committee of POSTECH<br><br>The following mice were used and maintained on a C57BL/6 background for experiments: WT C57BL/6; KN2/KN2; CD45.1 (B6.SJL-Ptprca Pepcb/BoyJ); OT-II (B6.Cg-Tg(TcraTcrb)425Cbn/J); Icosl-/- (B6.129P2-Icosltm1Mak/J); CD4-Cre (B6.Cg-Tg(Cd4-cre)1Cwi/BfluJ); and Bcl6-floxed (B6.129S(FVB)-Bcl6tm1.1Dent/J) mice.<br><br>The following mice were maintained on a BALB/c background for experiments: BALB/cJ; BALB/cByJ; BALB/cAnNCrl; KN2/KN2; Zsgreen-Tbx21; Rag1-/- (C.129S7(B6)-Rag1tm1Mom/J); Cd1d-/- (C.129S2-Cd1tm1Gru/J); Il4ra–/– (BALB/c-Il4ratm1Sz/J); CD45.1 (CByJ.SJL(B6)-Ptprca/J); DO11.10 (C.Cg-Tg(DO11.10)10Dlo/J) mice.<br><br>Lymphocytes were usually analyzed from 6-9 week-old mice. Regarding experimental purpose, thymi were isolated from embryonic day 15.5 (Figure S6) or 1 day-old newborn mice (Figure 3B and 3C), or 1-2 week-old mice (Figure 2A and Figure 3B). Both male and female mice were used for analysis.<br><br>KN2/KN2 and Zsgreen-Tbx21 reporter mice were previously described (Ref #11), and germ-free (GF) and antigen-free (AF) mice were maintained as previously shown (Ref #48). OT-II, Iocsl-/-, CD4-Cre, Bcl6-floxed mice were provided by Sin-Hyeog Im, and MD4 mice were received from Yoontae Lee.<br><br>In experiments using CD4-Cre Bcl6 fl/fl mice, litermate controls were bred in same cages. WT control mice were bred separately when analyzing Cd1d-/- BALB/c, Il4ra-/- BALB/c, Zsgreen-Tbx21 BALB/c, DO11.10, OT-II, Icosl-/- B6, or KN/KN2 B6 and BALB/c mice. Both female and male mice were used in experiments. Experimental mice were euthanized by carbon dioxide to isolate intact thymi. All animals were bred and maintained in a specific pathogen-free (SPF) conditions with exception of GF and AF mice used in Figure 2F.<br><br>All housing condition including SPF, GF, and AF condition provide ambient temperature 22~24'C, humidity 40~60% and a dark/light cycle of 12 hours.
Wild animals	No wild animals were used in this study.
Field-collected samples	No field collected samples were used in this study.
Ethics oversight	All experiments were approved by the Institutional Animal Cared and Use Committees (IACUC) of the Pohang University of Science and Technology (POSTECH).

Note that full information on the approval of the study protocol must also be provided in the manuscript.

Flow Cytometry

Plots

Confirm that:

- [x] The axis labels state the marker and fluorochrome used (e.g. CD4-FITC).
- [x] The axis scales are clearly visible. Include numbers along axes only for bottom left plot of group (a 'group' is an analysis of identical markers).
- [x] All plots are contour plots with outliers or pseudocolor plots.
- [x] A numerical value for number of cells or percentage (with statistics) is provided.

Methodology

Sample preparation	For analysis of IgE-expressing plasma cells, thymus were harvested from mice using mechanical dissociation through a 70µm cell strainer, washed and resuspended in RPMI medium plus 10% (vol/vol) FBS. The number of cells and viability were determined by an automated cell counting (Beckman Coulter Vi-CELL). Cells were stained with CD138-Biotin on ice for 30 min. After washing, they were incubated with streptavidin-conjugated microbead on ice for 30 min, and then washed. Afterward, CD138-expressing cells were enriched by MACS. CD138 enriched thymocytes were stained with fluorochrome-conjugated antibody cocktail for surface markers at 4°C for 30 min. Cells were subsequently washed with FACS buffer (DPBS +1% FBS). For intracellular transcription factor, immunolgoublins, and cytokine staining, we used eBioscience kit (#00-5523-00) and followed by manufacturer's instructions.<br><br>To isolate lamina propira cells from the intestine, harvested small intestine were minced by McIlwain Tissue Chopper and incubated with FACS buffer with 10 mM EDTA at 37°C for 20 min to remove epithelial layer, and then washed. And then, they were digested in 5 mL of RPMI-1640 containing collagenase D (400 Mandl Units; ROCHE) and DNase I (1 mg/ml; 9003-98-9) on a shaker at 37°C for 45min, followed by filtration through a 70 µm strainer and 40%, 70% Percoll (Merck) gradient centrifugation (20 min at 2,000 rpm at room temperature).

	To analyze mast cells in the ear skin, he ears were excised and cut into small pieces. The ear epithelial cell layer was removed by vigorous stirring in PBS containing 3% FBS, 20 mM HEPES, 100 U/ml penicillin, 100 µg/ml streptomycin, 1 mM sodium pyruvate, and 10 mM EDTA at 37°C for 20 min. The tissue samples were then digested in PRMI containing collagenase type V (1 mg/ml, Sigma) at 37°C for 45 min.
Instrument	LSR II (Becton Dickinson) and Moflo-XDP (Beckman Coulter) for cell analysis and sorting, respectively.
Software	Flowjo software v9 and v10 were used to collect and analyze data.
Cell population abundance	For single-cell RNA sequencing, dump (CD3, CD4, CD11b)-CD19+CD138+ PCs and B220+CD19+ B cells from thymus were sorted with 95-99% purity as we shown in Figure S8A.<br><br>For the adoptive transfer experiment, we used CD19+CD138+ PCs from spleen, mesenteric lymph nodes and Peyer's patches with 99% sorting purity as presented in Figure S7B.<br><br>For the FTOC experiment, we sorted mature B cells, CD4 T cells and NKT2 cells using specific surface markers with 93-98% purity as shown in Figure S6.
Gating strategy	For all experiments, we identified cells by gating on lymphocytes using FSC-A and SSC-A. Doublets were excluded using SSC-A and SSC-W. Surface CD3, CD4 and CD11b+ cells were gated out, and CD138 and CD19 double positive cells were used for PC sorting.

☒ Tick this box to confirm that a figure exemplifying the gating strategy is provided in the Supplementary Information.
